# Telemedicine, e-Health, and Digital Health Equity: A Scoping Review

**DOI:** 10.2174/0117450179279732231211110248

**Published:** 2024-02-06

**Authors:** Donatella Rita Petretto, Gian Pietro Carrogu, Luca Gaviano, Roberta Berti, Martina Pinna, Andrea Domenico Petretto, Roberto Pili

**Affiliations:** 1 Department of Education, Philosophy and Psychology, University of Cagliari, Cagliari 09124, Italy; 2 IERFOP Onlus, *Via* Platone 1/3, Cagliari 09100, Italy; 3 Global Community on Longevity, Italy

**Keywords:** Equity, Digital health equity, Telemedicine, e-health, Scoping review, Healthcare domain

## Abstract

**Background:**

With the progressive digitization of people's lives and in the specific healthcare context, the issue of equity in the healthcare domain has extended to digital environments or e-environments, assuming the connotation of “Digital Health Equity” (DHE). Telemedicine and e-Health, which represent the two main e-environments in the healthcare context, have shown great potential in the promotion of health outcomes, but there can be unintended consequences related to the risk of inequalities. In this paper, we aimed to review papers that have investigated the topic of Digital Health Equity in Telemedicine and e-Health [definition(s), advantages, barriers and risk factors, interventions].

**Methods:**

We conducted a scoping review according to the methodological framework proposed in PRISMA-ScR guidelines on the relationship between Digital Health Equity and Telemedicine and e-Health *via* Scopus and Pubmed electronic databases. The following inclusion criteria were established: papers on the relationship between Digital Health Equity and Telemedicine and/or e-Health, written in English, and having no time limits. All study designs were eligible, including those that have utilized qualitative and quantitative methods, methodology, or guidelines reports, except for meta-reviews.

**Results:**

Regarding Digital Health Equity in Telemedicine and e-Health, even if there is no unique definition, there is a general agreement on the idea that it is a complex and multidimensional phenomenon. When promoting Digital Health Equity, some people may incur some risk/s of inequities and/or they may meet some obstacles. Regarding intervention, some authors have proposed a specific field/level of intervention, while other authors have discussed multidimensional interventions based on interdependence among the different levels and the mutually reinforcing effects between all of them.

**Conclusion:**

In summary, the present paper has discussed Digital Health Equity in Telemedicine and e-Health. Promoting equity of access to healthcare is a significant challenge in contemporary times and in the near future. While on the one hand, the construct “equity” applied to the health context highlights the importance of creating and sustaining the conditions to allow anyone to be able to reach (and develop) their “health potential”, it also raises numerous questions on “how this can happen”. An overall and integrated picture of all the variables that promote DHE is needed, taking into account the interdependence among the different levels and the mutually reinforcing effects between all of them.

## 1 INTRODUCTION

The first definition of health equity described “health inequities” as “unnecessary, avoidable, unfair and unjust differences in health” [1]. Some years later, Braveman and Gruskin [2] discussed some general issues in the previous definition and proposed “health equity as the absence of systematic disparities in health or the major social determinants of health between groups with different levels of social advantage/disadvantage” [2]. In 2006, Whitehead and Dahlgren claimed that “equity in health should imply that virtually everyone could attain their full health potential and that no one should be disadvantaged from achieving their potential because of their social position or other socially determined circumstance” [3, 4]. In recent years, international interest in this topic has increased, thanks to the documents of the World Health Organization that underline the importance of guaranteeing all people the opportunity to develop their health potential, the importance of reducing the risk that someone may be disadvantaged, and the need to promote interventions aimed to increase “health equity” [5-7].

With the progressive digitization of people's lives and in the specific healthcare context, the issue of equity in the healthcare domain has extended to digital environments or e-environments, assuming the connotation of “Digital Health Equity” (DHE). In the documents of 2021 and 2022, the World Health Organization defines “digital health” and therefore focuses on DHE, thus defining an action strategy on digital health that recognizes the centrality of promoting equity and preventing the risks of discrimination and marginalization [6, 7].

In this general framework, Telemedicine and e-Health represent the two main e-environments in the healthcare context where the risks of inequity could be described [8]. Telemedicine refers to the provision of healthcare in situations where the health professional and the patient are not in the same physical location; in clinical-diagnostic-therapeutic evaluation, it is a clinical path in which digital remote interactive communication is established. Communication between the patients and the health professionals is activated, and data and information are moved [9, 10]. The patients are in their homes or at other sites, and they are in a different place from the clinicians; in this regard, it is also called “remote patient monitoring”. Although it is sometimes considered as a sort of synonym, the concept of e-Health, on the other hand, refers to a broader concept than Telemedicine as it refers to the more general use of technologies in the healthcare context [5-7]. Since the 1990s, this term has been used to describe the use of technologies and the Internet to enhance or provide access to knowledge and services in healthcare settings. Today, this concept has been extended to include the experience of all the “actors” involved and to include services, products, processes, and all the infrastructures involved in digitization in the healthcare sector [8].

Although the digitization process was slow and gradual in the last decade, in the last three years, this process has had a sudden and quick acceleration during the COVID-19 pandemic; the methods and tools of Telemedicine and the use of e-Health have spread to guarantee a continuity, albeit partial, in the provision of health services [9-11]. All this has been possible thanks to an emergency modification of the methods and regulations previously defined and this has made it possible to highlight virtuous phenomena and positive processes, and also to discover possible risks [8-15]. Innovations in digital health, Telemedicine, and e-Health have shown great potential in the promotion of health outcomes, but there can be unintended consequences related to the risk of inequalities [16]. Now, in a new phase after the COVID-19 pandemic, previous emergency experiences allow us to have some useful discussions on the progressive digitization process. These discussions are particularly relevant in the context of the so-called risk of the “digital paradox” of Telemedicine; it means that people could have better support from the digitization process, but they can also incur a high risk of difficulty in accessing services and information, and they can have a higher risk of exclusion from Telemedicine if all the elements and variables that can influence the use and the access to the processes, products and environments of Telemedicine are not correctly taken into account [17-19]. The current use of Telemedicine and e-Health has highlighted even more the need to better understand how to promote equity and how to prevent marginalization; thus, a general question arises: could the progressive digitization in health contexts help in the reduction of inequalities and the promotion of equity, or could it lead to an exacerbation of inequalities?

Keeping in mind these aspects, in this paper, we have aimed to review the papers that have investigated the topic of DHE in Telemedicine and e-Health and discuss the following research questions:

1) How did previous papers define and describe DHE in Telemedicine and e-Health?

2) How did previous papers describe barriers and risk factors in the promotion of DHE in those e-environments?

3) How did previous papers describe the advantages of the use of Telemedicine and e-Health for the promotion of DHE?

4) How did previous papers describe ways to improve equity in e-Health and Telemedicine?

## 2 MATERIALS AND METHODS

### 2.1 Protocol and Study Design

**Fig. (1):**
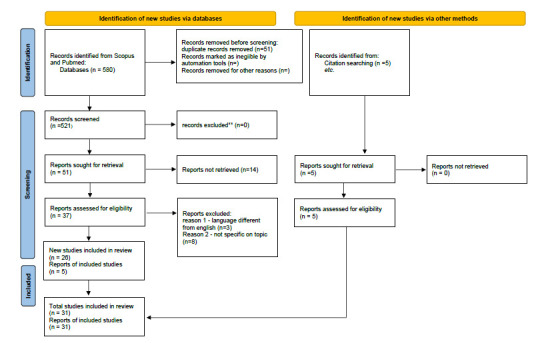
PRISMA 2020 flow diagram for systematic reviews resulting from a search of database and other sources for scoping review.

The protocol was developed using the scoping review methodological framework proposed in PRISMA-ScR guidelines [20] (Fig. **1**). We have reported data according to these guidelines.

We have conducted a literature review on the relationship between DHE and Telemedicine and e-Health *via* Scopus and Pubmed electronic databases. The following inclusion criteria were established: papers on the relationship between DHE and Telemedicine and/or e-Health, and written in English. All study designs were eligible, including those that utilized qualitative and quantitative methods, methodology, or guidelines report, except for meta-reviews. We have excluded papers written in other languages than English. Considering the novelty of the topic, no time limits have been considered.

### 2.2 Data Search

Literature search was conducted by two authors (DRP and GPC) in the following online databases: Scopus and PubMed. These databases were chosen to cover health sciences. We have used the following search keywords: DHE combined with the “AND” Boolean operators and “Telemedicine” combined with the “OR” Boolean operators, and “e-Health”.

### 2.3 Study Selection

The literature was selected, and the results have been analyzed. According to the needs, the keywords were searched in the publication title or abstract. A total number of 580 records was found. Two authors (DRP and GPC) independently reviewed the chosen references, deciding to exclude further papers and remove duplicate references. A total number of 51 papers was found.

### 2.4 Data Extraction

Papers were analyzed with respect to their content, and papers with content that was not fully within the scope of this review were eliminated. A group of 37 full-text articles were considered. Starting from the references in the full text of the articles derived from the literature review, some other papers were included (5 articles). After the reading of the full-text, a total of 31 papers were then considered for the final analysis.

### 2.5 Quality and Risk of Bias Assessment

According to Tricco and colleagues [20] and considering the peculiarity of the scoping review, we did not appraise the methodological quality or risk of bias of the included papers.

## 3 RESULTS

After examination of the included articles and according to the quality of the studies and the research questions, we did both quantitative and qualitative analyses of the papers according to the proposed research questions. Then, we synthetized and grouped the papers according to the four research questions. The findings have been summarized using a narrative and systematic review. Table **1** describes the 31 papers that have met the selection criteria.

As expected, according to the novelty of the topic, the included papers have revealed that only recently, the authors have addressed the themes of the review. The selected articles have been published in the last five years, mainly in the last 3 years, and only one article has been published in 2018 [21]. The geographical distribution of the papers has suggested a prevalence of interest in the USA [14, 17, 22-37], Canada [15, 21, 38-40], and in Australia [9, 41, 42]; one paper has been from Korea [43], and only some authors from Europe have addressed the topic [44, 45]. The papers have been mainly editorials [21, 22], commentaries [27, 36, 38, 46], or viewpoint papers [14, 23, 29, 39], and only a few research articles have focused on the themes of this scoping review [12, 17, 24, 25, 41, 44]. According to the digital e-environments or e-platforms considered, even if all the papers refer to Telemedicine and/or e-Health, some other fields are considered, like Telehealth [13, 14, 17, 22, 24, 25, 34, 36, 42] and mobile health [14, 21, 26, 37]. Some authors have focused their attention on health data and described the role of electronic health records [28, 31, 43], electronic medical records [28], personal health records [43], and patient-generated health data [43] in promoting DHE.

In the following, we have briefly described the findings from the sorted papers according to the main research questions.

### 3.1 How Did Previous Papers Define and Discuss DHE in Telemedicine and e-Health?

To date, there is no complete agreement on the definition of DHE, although the aforementioned documents of the World Health Organization represent a common thread between the different positions proposed by the authors [5-7]. However, the main shared elements among all the most recent definitions seem to be the promotion of one's health potential through digital tools [24, 35, 44], the reference to a list of risk factors [25, 38, 42] or to single risk factor [13, 27] that can lead to some forms of marginalization, and the reference to the description of group/s of people at higher risk of inequalities [12, 13, 15, 17, 21-24, 27-30, 32, 34, 35, 40-49].

Regarding “health potential”, only some papers refer directly to it, while other papers have been found to use related words like “full potential” [35], “optimal health” [24], and “greatest standard of health” [27]; sometimes the idea of “health potential” is considered only indirectly, like in the paper of Kaihlanen and colleagues (“digital inequalities may in turn cause significant disadvantages, such as an increased risk of health deterioration”) [44], or in the paper of Foley and colleagues that highlighted the idea that “benefit from knowledge and practices related to the development and use of digital technologies (may) improve health” [41]. Also, Crawford and Serhal chose a similar approach and focused on “poor health outcomes” (“unexamined inequities in access to and implementation of digital health can recapitulate and deepen the inequalities that have long existed within our health care system, and they can contribute to poor health outcomes”) [39].

Regarding the description of group/s of people at higher risk of inequalities, while the World Health Organization’ documents make a general reference to the concept of disadvantage, there is a common reference to a set of people or groups that could incur in forms of marginalization and could meet some barriers in the development of their health potential [5-7]. Those people are sometimes described in a general way as “groups of people with reduced resources” [23, 30, 36, 42], or as “marginalized group/s or individuals” [15, 21, 22, 28, 38, 46]. Some papers have described in a deeper way the group/s of people that could incur some forms of inequity (people with disability, people living in lower socio-economic areas, cultural ethnical, and economically diverse communities, elderly people, and people who live in rural areas) [27, 40]. As a general view, some authors converge in identifying social, linguistic, cultural, geographical, and health factors that can be associated with a greater risk of lack of equity of access (albeit with interesting differences between authors). Some papers have focused on specific reasons, like low digital literacy skills [42], while other papers have described a longer list of possible reasons, proposing a more general description of a “complex causal model” [27, 31-33]. Other papers have also described other general or specific reasons that can promote inequities, like social vulnerability, complex health problems, language barriers [42], and low financial and economic resources [17, 25].

Some authors have highlighted the multidimensional nature of DHE (individual level, contexts, social determinants of health, and the enabling environment) and the need to consider different levels to achieve a comprehensive knowledge of it [31, 38]. Anaya and colleagues [22] took a further step forward in defining the multidimensional features of “equity in digital healthcare” in Telemedicine, highlighting its complexity. Richardson and colleagues [31] proposed a general model where so-called social determinants of health (SDOH) [2, 47] are considered to contribute to the promotion of DHE, and they also proposed to integrate the SDOH with the so-called digital determinants of health (DDOH) at individual, interpersonal, community, and societal levels [31].

### 3.2 How Did Previous Papers Describe Barriers and Risk Factors in the Promotion of DHE in those e-environments?

Barriers and risk factors in the promotion of DHE in Telemedicine are discussed considering both the perspective of the users/individuals and the perspective of the professionals.

When the users’ perspective is considered, authors have described the following main barriers and risks: limited availability of devices, limited access, limited knowledge and previous experiences in the use of devices, tools, and e-environments, and language barriers, like those related to low English fluency [22, 33, 38, 40, 41, 43, 44].

Other kinds of barriers are described from the perspective of the users, like the one related to the need to have private space in the home (or in other facilities) with the aim to guarantee privacy and confidentiality during Telemedicine consultations [44]. Other authors have focused on the role of trust and confidence of users [28, 41] and lack of interest and motivation [34] in the effective use of instruments and tools of Telemedicine.

Some papers have described the role of formal and informal social networks and the effects of isolation on the access to information and tools needed to use Telemedicine tools and e-environments [41] and on the availability of help and support if needed [44]. The role of poverty and low income in the availability and access to e-environments has also been considered [41, 42].

Some authors have focused on the risks from the perspective of healthcare professionals, describing the point of contact with users (like the risk of the digital divide, mainly on the first two levels according to Shaw and colleagues [15], availability of devices and software, and experiences and specific knowledge in the use of e-platforms and e-environments) [15, 17].

Some peculiar risks have also been described for healthcare professionals; they can meet some difficulties in performing clinical skills through the tools of Telemedicine (devices, software, e-platforms, and e-environments) and they can need some specific training and/or support to transfer their skills on those tools and e-environments [8].

Although a lot of papers have discussed various barriers in access to digital health e-environments, only some authors have proposed an integrated and multilevel description of them [31, 33, 44, 47].

In some papers, barriers of DHE and inequities are used as synonyms for “digital divide” [15, 27]. Some papers have described the relationship between DHE and the digital divide according to two different approaches: firstly, defining “digital divide” as a general construct [27], and secondly, defining “digital divide” in a deeper way [15, 38]. Shaw and colleagues proposed an integrated definition of the digital divide, the so-called “three levels of digital divide”: the first level refers to the access to digital tools/processes, the second level refers to the knowledge/skills necessary to access tools and digital processes, the third level refers to the possibility or not of using digital tools and digital processes to obtain useful results for one's life [15, 38]. In the first level, people may have difficulty accessing Telemedicine services due to availability/not availability of devices, software, and Broadband access. In the second level, people may have difficulty accessing Telemedicine services due to language difficulties and/or barriers (English-speaking proficiency or need of an online translator or an interpreter), or limited knowledge and experiences in the use of computer and/or the required software and/or the required e-platform. In the third level of the digital divide, people may have the devices and knowledge needed to access Telemedicine services and e-health, but they are not ready to use digital tools and digital processes to obtain useful results for his/her own life. Shaw and colleagues [15] focused on the perspective of the users, but a similar approach has been discussed also considering the health professionals’ perspective [11, 17].

### 3.3 How did Previous Papers Describe the Advantages of the Use of Telemedicine and e-Health for the Promotion of DHE?

There is a general agreement on the idea that Telemedicine can produce overall advantages. The main advantage is to maintain access to care, continuity of care, and communication between patients and health professionals in critical and emergency situations, like the one related to the COVID-19 outbreak [17, 24, 27, 39, 41]. Some authors have described positive effects for users/patients: it can reduce general and specific costs [27], like those related to transportation and the loss of working hours, and it can reduce the time needed for a visit [22]. Some authors have focused on the positive effects on patients’ satisfaction, engagement, and communication with clinicians [24]. Some papers have described the positive effects on safety and health outcomes and the care of people with complex care needs and several illnesses and disorders [27, 45].

When the positive effects on health professionals have been considered, engagement and communication are described, and the reduction of workload is discussed (even some papers have also described the risk of an increase in the professionals’ workload) [47].

Regarding e-Health, there is a general agreement on some overall advantages, like improving access to health-related information [27]; even some authors have discussed the risk of spread of “fake news” and/or inaccurate information in the health field [45].

Regarding health data, Foley and colleagues [41] have highlighted the positive effects of gathering, tracking, and delivering health-related information for individuals and populations (even some papers have described some risks in this regard related to privacy and other issues in data management).

### 3.4 How did Previous Papers Describe Ways to Improve Equity in e-Health and Telemedicine?

There are two general approaches currently suggested by the authors who have dealt with these topics for the promotion of DHE: firstly identifying single areas of intervention and single levels of action, and secondly proposing general models of approaches.

In the first approach, individual areas for improvement are identified at the individual, community, and health policy levels. For example, Anaya and colleagues, in 2022, referred to the importance of improving the digital knowledge and skills of possible users of Telemedicine services, as well as improving the availability of the necessary infrastructures (including the availability of devices, hotspots, and dedicated spaces) [22]. Chang and colleagues [17] proposed some policy recommendations focused on economic issues of Telemedicine: reimbursement of telehealth visits when delivered by telephone and expanding free Broadband access. Lopez de Coca and colleagues proposed specific interventions aimed to reduce the risks of fake news spread with e-Health portals and e-environments [45].

The paper of Saeed and Masters (2021) focused on the need to improve internet access and educate/support patients in the use of technologies, tools, and software, and to derive benefits from these technologies in their lives [33].

The second approach also includes the work of Shaw and colleagues in 2021 [34], where there have been identified three general areas of strategies useful for promoting equity in Telemedicine and in digital health contexts: simplifying complex interfaces and information flow, using intermediaries, and creating mechanisms through which people at risk of disadvantage in accessing digital healthcare can provide useful inputs for the design and implementation of Telemedicine interventions [34]. The same authors have also identified three different levels of intervention for the promotion of DHE: political and managerial level to guarantee the presence of the required infrastructures and allow the reimbursement of services to facilitate access to all segments of the population; at the level of health services for monitoring the quality of services and their actual usability by groups at greater risk of discrimination in order to ensure the training of the various health professionals involved; at the level of the community and of individual patients for direct involvement in the various phases and for the implementation of interventions aimed at increasing the level of basic and more sophisticated digital knowledge and skills, useful for being able to use Telemedicine tools and services [34]. Gallegos-Rejas and colleagues [42] have also described strategies and tools that can be used to reduce the risk of inequity in digital healthcare, focusing in particular on overcoming some barriers experienced by people in terms of the digital divide (increasing awareness of the gap in knowledge in the technological field and in digital literacy between professionals and service users, promotion of training courses, promotion of interventions aimed at increasing useful knowledge for health professionals and for device and software manufacturers to allow progress in design, implementation and use of Telemedicine services, promotion of reflections and interventions aimed at increasing equity of access and usability, and taking into account individual differences in digital literacy) [42]. Brewer and colleagues, in 2020, focused on the design and implementation tools and methods that involve the direct involvement of communities and of those groups that could incur the greatest risk of exclusion [23]. Lyles and colleagues [47] agreed on a similar approach; in order to avoid the risk of a lack of equity in access and usability in the health sector, it is very important to take these aspects into account right from the planning, implementation, and then monitoring stages of each intervention in the field of digital health [47].

Anaya and colleagues [22] hypothesized the creation of a “Telemedicine ecosystem”, which represents an example of the second approach, in which different levels are taken into consideration (both individual skills, the availability of infrastructures, and the different environmental conditions and economic and political support for the implementation and feasibility of the interventions). As a step forward, they have reflected on strategies that can be implemented to promote equity and limit the risk of marginalization and lack of use of people according to an approach that some authors call “creation of an ecosystem for the promotion of equity in Telemedicine” [22] and which requires the collaboration of all the actors involved in the process of designing, implementing, disseminating, and using Telemedicine systems.

Crawford and colleagues [39] referred to the “Digital Health Framework”, which integrates digital determinants of health and DHE in each implementation of digital health solutions and programs, enabling the direct involvement of people from “marginalized and vulnerable groups” in the position of digital health leadership or in co-designing at all stages of innovation and implementation.

A common approach among different papers is the reference to multiple levels of analysis and intervention: individual level, interpersonal level, community level, and societal level [31-33], and the awareness of the complex causal model [31].

## 4 DISCUSSION

This review has summarized the evidence regarding the promotion of DHE in Telemedicine and e-Health. On the one hand, some authors have recognized the role of Telemedicine and e-Health in reducing the gap in access to health services and promoting greater equity and opportunities for access. On the other hand, the same authors and other authors, however, have identified fields that could be improved. It is, therefore, quite clear that the diffusion of Telemedicine tools can increase the general possibilities of treatment for all people, and some advantages are well known to be strongly linked to the possibility of creating and maintaining certain conditions necessary to guarantee equity of access and use, also by virtue of the aforementioned acceleration of the diffusion process of Telemedicine during the COVID-19 pandemic, which has allowed its widespread diffusion in both primary and special care [8-15]. Some authors have also highlighted that the diffusion of Telemedicine can be considered as a sort of double-edged sword; on the one hand, Telemedicine can increase the possibility of accessing treatment, but on the other hand, the most vulnerable populations from both a socio-economic and health point of view, who could benefit the most from Telemedicine, could be those less ready to use it [29, 47], and this raises reflections on the potential inequalities of access that could result from the massive use of Telemedicine [26, 44], with reference to the so-called “inverse care law” [48].

In this paper, we have addressed four research questions to discuss these issues in a deeper way. The first research question has addressed the definition of DHE in Telemedicine and e-Health. Even if there is no unique definition, there is a general agreement on the idea that it is a complex and multidimensional phenomenon, where there are at least three/five different levels: the individual level, the interindividual level, the social level, the community level, and the institutional level, with interdependence among the different levels and the mutually reinforcing effects between all of them. There is also an agreement on the role of the so-called “enablement environment” that can promote or reduce DHE and the relationship between DHE and social determinants of health (SDOH/s). Some authors have introduced and described the so-called digital determinants of health (DDOH), which interact with SDHs in the promotion or reduction of DHE in Telemedicine and e-Health.

The second research question has focused on the risks and the obstacles in the promotion of DHE in Telemedicine and e-Health. There is a close link between the first and the second research questions because the description of risks and obstacles in the promotion of DHE is strictly correlated to the definition of DHE proposed by each paper. There is a general agreement on the idea that when promoting DHE, some people may incur some risk/s of inequities and/or they may incur some obstacles. While sometimes those risks/obstacles are described in a general way (categories of risks) or in a specific way (description of single risk), other approaches have described group/s of people that may incur some kind of risks/obstacles. The last approach may have inferred, as a negative consequence, that some groups of people could be considered as implicit “bearer” of some kinds of risks and obstacles.

The third research question has described the general and specific advantages of the use of these two e-environments in the health context to promote equity. There is a general agreement on the idea that there can be advantages and benefits both for users/patients and for health professionals, with differences and points of contact between them.

The fourth research question has analyzed the different approaches to improve equity in digital health through Telemedicine and e-Health described by the authors. There is a close link between the fourth and the second research questions because interventions aimed at promoting DHE are strictly related to the risks and obstacles described in each paper. The sorted papers have described the different approaches: firstly, an approach that addresses a specific field and level to improve equity, and secondly, an approach that proposes general and multidimensional model/s. According to the second approach, any intervention may consider all the levels and address all of them to guarantee their effectiveness in the promotion of DHE, taking into account the interdependence among the different levels and the mutually reinforcing effects between all of them.

This scoping review has some limitations related to the nature of the topic and the need for a better understanding of the complex causal process/es that can generate equity or inequity.

As has already been said, there is a broad international debate on the concept of “health equity”, and also on the concept of “equity” in a broad sense, from which derives the general awareness that by promoting equity in different life contexts and life domains of people, there may be some critical loci. If this awareness is in part increasingly shared, less shared is the discussion on those critical loci and on the complex causal process/es that can generate those critical loci. In other words, there is no agreement on the causal processes that can limit equity and, as a negative effect, promote “inequities” or “less equity”. There are two different approaches in the analysis of those causal processes. There is a general tendency to compare a process-based vision (which sees the “lack of equity” as the result of a negative interaction between the “person” and the “environment” in a broad sense, be it physical or social) with visions that see the “lack of equity” as an intrinsic risk to the person (almost a characteristic or an attribute of him/her, rather than the consequence of the interaction between this “person” and an “environment”, once again both physical and social). Because of this general tendency and in a similar vein to what happens in the field of disability, equity and lack of equity can also be perceived alternatively as “attributes of the person” or as “the consequence of a negative process of interaction between the person and an environment”. In the field of disability and the field of equity, these two visions tend to coexist, although the second is the one more consistent with current conceptualizations [49]. Unfortunately, even the first vision has a huge and negative effect, sometimes influencing the a priori definition of groups of people who in themselves “can/necessarily have to run the risk of less equity”, and thus generating processes of a priori categorization and stigmatization. This dichotomous vision, “process” *versus* “attribute”, can also have effects on the terminology or terminologies used: “processes for the promotion of equity and equal opportunities” in the first case, and groups of people as “bearers of less equity” (often labeled with terms, such as “marginalized” or “under-resourced) in the second case. This categorical approach inherently carries the risk that only little attention will be paid to the need to prevent the risks inherent in negative interactive processes and little attention will be paid to the promotion of virtuous interactive processes aimed to increase equity and support the promotion of one’s health potential. We deem it useful to point out that the second vision of inequities or less equity clearly has a procedural and dynamic value, also for intervention; it is not the single individual or group that incurs the risk of disadvantage, but the disadvantage is the consequence of the interaction between any person and an environment that is “not sufficiently equipped to promote health equity”.

## CONCLUSION

In summary, the present paper has discussed DHE in Telemedicine and e-Health. Promoting equity of access to healthcare is a significant challenge in contemporary times and the near future. The progressive digitization of healthcare and the dissemination of Telemedicine’s tools and methods currently represent some of the ways in which this challenge is being addressed; these being complex tasks is evident from the choice of the word “challenge”, which connotes their specificity and which stimulates a cautious attitude of analysis and study of all the variables involved.

While on the one hand, the construct “equity” applied to the health context highlights the importance of creating and sustaining the conditions to allow anyone to be able to reach (and develop) their “health potential”, it also raises numerous questions on “how this can happen”. On the other hand, the progressive digitization of the healthcare context also highlights further areas of discussion and analysis. There are some specific lessons learned during the current and previous use of Telemedicine and e-Health, also taking into account the emergency experience during the outbreak of COVID-19 when the e-environments became necessary to guarantee care and continuity in healthcare provision. Bearing in mind all those aspects, what may be the next steps in the understanding of this field of study, with the aim to increase DHE?

In agreement with the results of the scoping review, we believe it is useful to list the reasons/variables that can facilitate the occurrence of the condition of disadvantage and to consider all these variables with the aim of promoting equity and reducing risks of inequities. We agree with the need to have an overall and integrated picture of all these variables, a multilevel complex model of “Telemedicine and e-Health ecosystem”, like the ones proposed by various papers of this review and considering the interdependence among the different levels and the mutually reinforcing effects between all of them. Thus, health policymakers and health professionals should collaborate with communities and people to minimize the risk of inequity in access to health services and information and in the use of Telemedicine and e-Health. Moreover, government, scientific societies, stakeholders, and health policymakers may have a central role in planning and implementing specific interventions to promote health equity and DHE, providing system-level changes according to the chosen multilevel complex model of “Telemedicine and e-Health ecosystem”.

## AUTHORS’ CONTRIBUTION

D.R.P. and R.P. contributed to conceptualization; D.R.P., G.P.C., and L.G. designed the methodology; D.R.P., G.P.C., R.B., and L.G. conducted the literature review; D.R.P. and A.D.P. wrote the original draft; and D.R.P., R.P., A.D.P., L.G., R.B., G.P.C., and M.P. contributed to writing, review, and editing. All authors have read and agreed to the published version of the manuscript.

## Figures and Tables

**Table 1:** Papers which met the inclusion criteria.

**Author/s (year)**	**Country/s**	**Definition of Digital Health Equity**	**Description of People at Risk of Inequities**	**Kind of Study**	**Telemedicine (TM)/e-health (E)/TeleHealth (T)/ Digital Health (D)/Mobile Health (M)/electronic Health Records (EHR)/Electronic Medical Records (EMR)/personal health records (PHR)/ patient-generated health data (PGHD) Field/s (if declared)**	**Barrier/s and Risks in TM and E in the Promotion of Digital Health Equity**	**Advantages of TM and E in Promoting Digital Health Equity**	**Areas/levels of Improvement **
Anaya *et al.* (2022)	California, Los Angeles	Equity in telehealth primary care occurs when system infrastructure meets patients at their technological capacity and helps strengthen access to the system	Historically marginalized, low income, and limited English-speaking populations	Editorial	Telehealth Primary Care	- people can have limited access to computer and smartphones - people can have limited experience with computers and required software -telehealth platforms are English-only - some individuals may have lack of access to private space for virtual visits -some individuals may have limited Broadbent access	-reduced transportation barriers and barriers such as having to take time off from work for appointments - provides some convenient touchpoints that complement chronic care management in the traditional in-person settings	- telehealth ecosystem (Use of technology inclusive of economically marginalized patients, access to the technology and broadband for completing virtual visits and concrete support for patients as they develop their digital and telehealth skills) Six levels of policy recommendations for system level change to dismantle structural barriers limiting access in historically marginalized and low-income population (software, hardware, physical space, language compatibility and individual level)
Brewer *et al*., 2020 (Brewer *et al*., 2020)	Rochester, USA	The use of technology in digital health can cause differences in health for groups without may resources	groups without many resources/disadvantaged groups/ethnic minorities	viewpoint	E- Health Digital Health	-New configuration of digital divide (a paucity of culturally informed or culturally useful health information or digital health interventions) -a contextually developed innovations may benefit health outcomes in one sector of society while creating, sustaining or increasing health disparity in another (es. Pokemon Go, physical activity trackers)	New ways of working with health information and health care providers, including video doctor visits, text message reminders to take medicine and exercise and other ways of people to get their health information when and how they want and need it	-it is important that people and companies who develop these new technologies understand the challenges faced by disadvantaged groups -Innovation through community-engaged research -Development of best practices in strategic, design and implementation of health informatics and digital health intervention in marginalized communities
Chang *et al.* (2021) (Chang *et al.*, 2021)	New York, USA	Potential disparities in access to care arising from the widespread use of telehealth	-Historically underserved, low income communities -Social Vulnerabilities of the communities -medically and vulnerably populations	research	T Primary Care	- two forms of digital divide: one among health care providers (low reimbursement rates, lack of investment in telecommunication infrastructure, issues with interoperability, need to redesign workflows to accommodate telehealth, challenges around time management, technical issues) and the other among patients (limited access and limited digital literacy)	-maintaining critical access to care while keeping both patients and providers safe from unnecessary exposure to the coronavirus - maintain continuity of care by preserving the patient-provider relationship when in-person visits may not be feasible - patient portals can reduce administrative burden by allowing patients and providers to schedule appointments, communicate through direct messaging, and complete virtual prescriptions refill on their own time.	Three policy recommendations: - Reimbursement of telehealth - Reimbursement of telephonic visits on par with video visits - Expanding free Broadbent access -
Chaudhuri (2022) (Chaudhuri, 2022)	Calgary, Canada	The concept of digital health equity is complex and multidimensional. It integrated s comprehensive consideration of individual contexts, the social determinants of health, and the enabling environment	Marginalized populations, particularly those engaging the social gradient of minority ethnic communities	Commentary	E Virtual care	The risk that individual in the final level of digital divide, who have access to technology and possess digital literacy, in addition to having competencies in digital navigation, can be still not always able to achieve quality of outcomes	-	Transition from an exclusive service to an equitable standard
Cheng *et al.* (2020) (Cheng *et al.*, 2020)	Australia	Unequal access, vulnerable groups are at risk of being marginalized in the digital age Not everyone has the same access or skills to take advantage of the benefits and convenience of digital health	Vulnerable groups	Research	E D T	Digital divide Not everyone has the same access or skills to take advantage of the benefits of Digital Health	Telehealth as an important tool in providing patient consultations and treatment (during lockdown)	Ophelia (Optimizing Health literacy and access) is a co-design approach, a method for co-creating solutions to improve access, equity and outcomes by addressing literacy needs User involvements
Chen *et al.* (2022) (J. Chen *et al.*, 2022)	Maryland, USA	-Promote of health of all the people in all communities by strengthening, supporting and mobilizing communities and partnerships to improve health - promoting policies, systems and overall community conditions enabling optimal health for all	-Vulnerable patients with complex health problems -racial and ethical disparities in health care and health	research	T E Hospital settings and post discharge settings (HIT Health information technology)	Systemic and structural barriers	-HIT in post discharge as useful to connect patients with social services or community programs -data sharing - reduction of inpatient visits and readmissions - reduce structural racism and discrimination -improve patient’s engagement through customized design of patient portals and improve communication by reflecting patient’s cultural background and preferences - coordinate care, provide health care access and promote patients’ education	Horizontal multisector integration and vertical level integration to improve health care access, quality, reducing costs and improving health equity
Chunara *et al.* (2021) (Chunara *et al.*, 2021)	New York, USA	Healthcare disparities, healthcare technologies have the potential to exacerbate disparities via digital divide	-racial disparities - communities and populations of diverse race/ethnicities and sociodemographic due to social, language, financial and other barriers	Research	T	- risk of racism, sexism and ageism and their intersection -digital divide	- reduce healthcare disparities for patients in remote areas	- identifying where in the healthcare process disparities manifest is essential in order to inform effective programs, empower patients, and improve health outcomes
Crawford & Serhal. (2020) (Crawford & Serhal, 2020)	Toronto, Canada	Unexamined inequities in access to and implementation of digital health can recapitulate and deepen the inequalities that have long existed within our health care system, and they can contribute to poor health outcomes.	Health inequities between communities and across the life course of individuals (racialized groups, poverty, under-resourcing of health systems and neighborhoods, homelessness, other factors that decrease engagement with technology and with digital health literacy skills	viewpoint	D	Digital health technologies interact with social, cultural and economic realities and with social determinants of health to indirectly contribute to health equity	Digital healthcare can ensure ongoing access to clinical care and allow public health measures	-Digital Health Equity Framework which integrates digital determinants of health and digital health equity to become mainstream in all implementations of digital health - meaningful involvement of people from marginalized and vulnerable group in positions of digital health leadership, as health providers and in codesign at all stages of innovation and implementation
Durocher *et al.* (2021) (Durocher *et al.*, 2021)	Ontario, Canada	Health equity as removing barriers to care services to help individuals reach optimal health, digital health inequities as digital divide	Communities and groups that may be vulnerable due to factors such as location, age, health literacy and socioeconomic status (older adulthood, racialized communities, people who live in rural areas and people who have a lower socio-economic status	Review	D	Digital divide, lack of access to digital resources for specific vulnerable communities and groups	The role of technology industry and individual companies in addressing issues that widen the digital divide through promoting access to health services, technology infrastructure and focusing on systems that impact the social determinants of health	Technology industry’ initiative to improve digital health access - Provision of technology infrastructure and devices - Education programs - Transportation services - Disease management - Vaccination programs - Banking access - Technological development of no-profit organizations - Campaigns and fundraising - Housing projects - Back to work programs Healthcare and technology industry partnership to enhance access - Application technology development - Coalitions for health access - Low-cost services - Foundations - Developing health technology systems - Virtual care - Healthcare referrals - Disease specific initiative
Foley *et al.* (2021) (Foley *et al.*, 2021)	Australia	Digital health equity is concerned with fair and just access to, use of and benefit from digital health services and it is a critical axis of contemporary health promotion	Resources needed to benefit from Digital health services are distributed unevenly across society	Research	D	-Social isolation and poor health are associated with lower probability of internet use for health information -effective use of digital health services is less likely for people with low incomes or who are members of a minority ethnic group, followed by low levels of education, age, literacy, gender and rurality Less trust and less confidence	-Gather, track, deliver health-related information for individual and populations -Benefits from digital services for individuals	Theoretical framework Reference to social determinants factors, trust and e-health literacy Role of trust in predicting us of digital health services
Gallegos-Rejas *et al.* (2023) (Gallegos-Rejas *et al.*, 2023)	Queensland, Australia	Tele health inequity, telehealth can improve access to services to under-resourced communities, but ironically, the equity problem may be further compromised when telehealth is also difficult to access	Under-resourced communities People living in lower socio-economic areas, cultural and linguistically diverse communities, people living with disabilities, people with low health literacy	Focus	T	People living in lower socio-economic areas, cultural and linguistically diverse communities, people living with disabilities, and low health and/or digital literacy can experience difficulties accessing telehealth -The risk of digital divide (inability or access or benefit from emergency technologies (Ability to use a technological device (digital literacy) and to access to the technical infrastructure required to support telehealth services - cultural and language barriers	-Patient-centered design of care -Culturally appropriate solutions - trusted relations between care providers - confidentiality of patient information - policy-level changes to help with the uptake of telehealth services - involving of multiple stakeholders to implement effective and culturally competent telehealth solutions addressing equitable access to health care - cultural, linguistic, socio, spiritual needs of patients - promotion of digital literacy - and awareness of the gap in the digital literacy among consumers and telehealth professionals to promote co-design and user-engagement methods	Practical steps to reduce digital divide and encourage equitable access to telehealth. - awareness of the gap in the digital literacy among consumers and telehealth professionals to promote co-design and user-engagement methods - support to consumers, practitioners and health services managers and building capacity to design, implement and access telehealth services - adapted telemedicine- specific training program for practitioners and for consumers - to explore how Telemedicine-specific programs can include people with diverse digital literacy, to promote equitable access to telehealth
Hernandez and Rodriguez (2023) (Hernandez & Rodriguez, 2023)	USA	Health techequity/health disparities as a health difference that adversely affects disadvantaged populations, based on one or more than one health outcome Digital inclusion as the activities necessary to ensure that all individual and communities, including the most disadvantages, have access to and use of information and Communication Technologies	Race and ethnicity may be an independent contributor to health outcome	Review	D TM M Cardiovascular care	Certain populations experience barriers to Telemedicine access and decreased representation in cardiovascular digital health trials	Digital health interventions shoe incredible potential to improve cardiovascular diseases by obtaining longitudinal, continuous, and actionable patient data, increasing access to care, and decreasing delivery barriers and costs	-Promotion of digital inclusion, as the activities necessary to ensure that all individuals and communities, including the most disadvantages have access to and use of Information and Communication Technologies -multilevel intervention at individual, family, community, service s and policy level - representation of minoritized groups in all stages of the process (product development, clinical research and health services deployment
Jaworksi *et al.* (2022) (Jaworski *et al.*, 2022)	USA	Health disparities in digital health Digital divide Digital health justice, equitable opportunity for everyone to access, use, and benefit from digital health, to achieve the greatest standard of health and wellbeing	Persons with low socioeconomic status, racial, and ethnic minority groups and older individuals	Commentary	D	Digital divide	Improving access to health-related information and healthcare Reducing healthcare system inefficiencies, Improving quality of care Lowering healthcare costs Providing more personalized health care experiences	Centering equity in all digital health research, policies and practices Recommendations with the goals of: - Cultivate equity-focused behavioural and social science researchers - Improve access and use of digital health services to address disparities and promote digital health equity 1)Centering equity in research teams and theoretical approaches, 2)focusing on issues of digital health literacy and engagement 3) using methods that elevate perspectives and need of underserved populations 4) ensuring ethical approaches for collecting and using digital health data, understanding limitations, and mitigations biases 5) developing strategies for widespread adoptions and use of digital tools within and across systems and settings
Kaihlanen *et al.* (2022) (Kaihlanen *et al.*, 2022)	Finland	Everyone does not have an equal opportunity to benefit from digitization Digital inequalities may in turn cause significant disadvantages such as an increased risk of health deterioration Digital health equity as an equal opportunity for individuals to benefit from knowledge and practices related to the development and use of digital technologies to improve health.	Vulnerable groups Vulnerable positions Disadvantaged by health, economic, cultural or social conditions Older people, migrants, mental health service users, high users of health services and unemployed	Research	D	Vulnerable groups experienced problems with many digital determinants of health -Access to digital resources - inadequate and difficult to find support for service use - not having a eID for migrants - communication-related weakness -risks of securities and privacy vulnerabilities	Awareness of Digital determinants of health Digital health literacy	-Promotion of mitigating strategies, like increasing physical access, digital skills social support and improving digital remote support infrastructures -importance of hybrid strategies, including both high and low-tech perspectives and combination of online and offline strategies -better usability of digital services and on the opportunities and ability of individuals to benefits from them
Koele *et al.* (2022) (Koehle *et al.*, 2022)	USA	Social inequities Digital inequities Epistemic justice in digital health Centering equity in digital health means balancing improved reach with increased risk in digital health	Marginalized patients Vulnerable populations Vectors of power such as race and ethnicity, gender identity and modality, sexuality, disability, housing status, citizenship status and criminalization status	Review	D EHR/EMR	Reduced trust, alienation reduced access even services are theoretically available	-	Description of digital determinants of health and digital health equity Specific attention to health equity consideration in design, implementation, and evaluation Collaborations with users and patient groups to define priorities, ensure accessibility and localization, and consider risk in development and utilization of digital health tools Adoption of diversity, equity and inclusion (DEI)-related strategies
Lee *et al.* (2021) (Lee *et al.*, 2021)	Korea	Digital health equity/inequity, disparities in the quality of healthcare and treatment among population groups can be caused by unequal access to healthcare services, the digital divide and disparities in medical information	Populations groups (disparities in the quality of healthcare and treatment among population groups can be caused by unequal access to healthcare services, digital divide, and disparities in medical information)	Review	HER PHR PGHD	Differences in the accessibility, utilization capabilities and quality of technology, depending on the users’ characteristics Disparities in the quality of healthcare and treatment among populations groups can be caused by unequal access to healthcare services, the digital divide and disparities in medical information	Health-related information can be provided in consideration of individual circumstances and needs	General suggestions: -medical consumers should be engaged from the early stages of information production - digital literacy education and training should be provided over the long term - competent experts capable of producing easy-to-use and fun medical conte should be cultivated -medical information should be visualized and presented from the perspective of the user experience - a medical thesaurus should be created to help clarify confusing terms for users
Lisker *et al.* (2022) (Lisker *et al.*, 2022)	California	Digital health approach often lack a focus on health equity	Historically excluded groups Marginalized populations such as those with low income or with complex medical and social needs	Commentary	D TM	Multilevel barriers in technology access (barriers affecting interest, readiness, and digital literacy at the patient level, caregivers support and space for technology use at the family and home level, digital capacity and infrastructure at the community level, language concordant staff and digital training at the services level, broadband, devices and reimbursement at the policy level)	Technological innovation can improve health care and population health	Incubation completed raid-cycle research and evaluation projects with industry and in partnership with publicly insured patients and public health care workers to improve the fit of technology with diverse user needs and preferences Partnership between technology offerings and the needs and preferences of publicly insured patients
Lopez De Coca *et al.* (2022) (Lopez de Coca *et al.*, 2022)	Spain	Digital health divide	Digital divide Generation divide (over 70 years old) Geographic divide	Review	D TM E	Digital Divide Fake news, inaccurate information	Internet for healthcare purposes as an important solution to adequately meet the complex care needs of people with several illnesses	To reduce the risks of fake news, To ensure that digital tools are used correctly and competently To identify patients without devices or internet To create a common knowledge base To support accessible, easily navigable solutions To educate users To reinforce and improve patients-physician relationships To increase e-health literacy level, especially among the elderly population in order to avoid mismanagement of health information and direct it to more reliable sources
Lyles (2021) (Lyles *et al.*, 2021)	San Francisco	Achieving digital health equity entails not only ensuring n access to digital infrastructure but also designing digital health solutions with the broad range of end users in mind, implementing them in ways that address the unique needs of patients who require health-related safety-net services and evaluating their effects across a range of populations and health systems.	Black, Hispanic/Latin, lower-income communities	viewpoint	D	Troubling barriers to digital health access Structural deficiencies within the digital infrastructure but also designing digital health solutions with the broad range of users in mind	-awareness of digital literacy, interest and readiness in single individual, Digital capacity and infrastructure need in the community, digital training and technical assistance in professionals,	Focus on usability and relevance Design for multiple contexts Codesign with community Implement and evaluation in clinical settings Improve connectivity, improve accessibility, change of reimbursement policy
Mc Call *et al.* (2022) (McCall *et al.*, 2022)	USA	Digital Redlining, the systematic process by which specific groups are deprived of equal access to digital tools such as internet, that creates inequities in access to educational and employment opportunities, as well as healthcare and health information	-Under-resourced communities -Black and brown communities Low income communities Rural communities Elderly with low digital literacy Groups who have been historically marginalized	Perspective	Broadbent internet access to healthcare	Digital divide Digital redlining	Access for people working remotely, online and distance learning, virtual health visits via video or phone	Social-ecological model as a roadmap to address digital redlining and expand broadband access to communities, as a framework that acknowledges multiple levels of influences: - Societal strategies (expand internet infrastructure and access, redefine broadband, regulatory policies) - Community strategies (increase financial support fort under-resourced schools, access to equitable health platforms) - Relationship strategies (employ community-based digital navigators, increase awareness of and access to digital navigators) - Individual strategies (increase access to digital devices, increase digital literacy)
Ramsetty and Adams (2020) (Ramsetty & Adams, 2020)	Charleston, South Carolina	Technology may actually be widening the gap between groups both nationally and event globally due to persistent social, economic and political factors Digital divide can perpetuate inequity based on various social factors	Underserved populations	Perspective	Telehealth platforms	Digital divide Many patients could not access to the online system	During Covid19 outbreak, immediate adaptation of clinical care delivery system	During transition to telehealth based care, to put measures into place to ensure that patients did not lose their access to health care
Richardson *et al.* (2022) (Richardson *et al.*, 2022)	USA	Health equity refers to the absence of health inequities, differences in health that are unnecessary, avoidable, unfair and unjust	Several defined populations, including rural populations, persons with low incomes, racial, and ethnic and sexual and gender minorities	Review	EHR D Patient portals	Systemic oppression as root cause of disparities Differences in successful telehealth use in disparity populations Social. Determinants of health and Digital determinants of health	The development of patients’ portals allows patients online access to key elements of their medical charts	Comprehensive framework for digital health equity With digital determinants of health: -Individual level -interpersonal level -Community level -societal level
Rodriguez et al., (2022) (Rodriguez *et al.*, 2022)	Massachusetts	Digital inclusion as activities necessary to ensure that all the individuals in communities, including the most disadvantages, have access to and use of digital tools. Digital redlining	Disparities in portals use based on age, race, socioeconomic status, English-language proficiency, and other factors	Perspective	Digital Health tools such as T and patient portals	Structural barriers to digital inclusion, like digital redlining (discrimination by internet service providers in the deployment, maintenance, or upgrade of infrastructure or delivery of services -Correlation between redlining and poor health outcomes -limited Broadbent infrastructure, lack of access to internet-enabled devices	-	Community-based approach Digital health initiatives for digital literacy programs Infrastructure initiative (broadband infrastructure, broadband and device affordability) Promote digital literacy and evaluate impact of digital-infrastructure initiatives on health care disparities to guide future investment and policies related to digital inclusion
Rodriguez *et al.* (2020) (Rodriguez *et al.*, 2020)	Massachusetts	Digital health equity	Underserved populations (including rural populations)	Viewpoint	T Patient portals Mobile health apps	Digital divide Digital health literacy Inclusive design	Patients’ access to their health data Health data more computable and give patients more control on their medical records	Creating a more equitable technology landscape in health care requires a multifaceted approach to policy and design Closing digital divide, supporting technology access and use of digital tools Inclusive design Various stakeholders (governmental agencies, vendors, institutions, clinical teams, patients)
Roy *et al.* (2022) (Roy *et al.*, 2022)	USA	Equity according to WHO is the absence of unfair, avoidable, or remediable differences among groups of people, whether those groups are defined socially, economically, demographically or geographically or by other dimensions of inequality	Sex, gender, ethnicity, disability or sexual orientation	viewpoint	TM T M E	Different definitions of TM and T in different countries, state and also at a organization’s level	During Covid-19 outbreak, the role of telehealth shifted from an option to a necessity to maintain access when in-person care was deemed too risky	The need of a shared definition of TM and T to increase equity and clarity to support clear communication and interoperability and to support patients
Saeed and Masters (2021) (Saeed & Masters, 2021)	USA	Disparities as differences in treatment between racial, ethnic or other demographic group that are not directly attributable to variations in clinical needs or patients’ preferences and persist even after adjustment for socioeconomic status	Minority subpopulations	Review	T Patient portals	Digital divide (caused by poverty, low literacy, lack of interest and motivation to use technology, lack of access to technology) Role of social determinants of health	Use of technology in health care to improve outcomes Health information Technology offers a potential to expand access to health care, enhance clinical outcomes, and improve quality of health care	To Improve internet access To provide it support To advert technologies to patients To educate patients on the benefits of these technologies
Shaw *et al.* (2021) (Shaw *et al.*, 2021)	Canada	Digital divide and three levels of digital divide: -First level (to have/to not have access to technology) -Second level (disparities in technology literacy) -third level (if even people have enough competencies to use technologies, they might not be able to convert their use of technologies into outcomes that improve their lives)	Marginalized communities (including low-income and racial and ethnic minority groups)	Review	Virtual care programs Patient portals	Digital divide: -First level divide (disparities in access to technologies) -Second level (disparities in technology literacy) -Third level (disparities in outcomes related to technology use)	During Covid-19 outbreak, the primary strategy for maintaining access to ambulatory and outpatient health services has been to rapidly virtualize, creating systems of health care that rely on telephone visits, video visits, and methods of asynchronous communication such as email, SMS, text messages, and patient portals messages	Three levels of intervention: -policy and government, - organization and health system - community and patients. Three strategies: -To simplify complex interfaces and workflows -To use supportive intermediaries -To create ways through marginalized community members can provide immediate input in the planning and the delivery of virtual care
Sinha and Roy (2018) (Sinha & Schryer-Roy, 2018)	Canada	Digital health in its engagement with complex social settings can have diverse impacts – positive, negative, and mixed- on equitable health services and gender relations in communities	Low- and middle-income countries Marginalized and vulnerable groups	Editorial	Digital health E-health Mobile health	The absence of a strong health equity and gender analysis when designing, implementing and evaluating digital health policies and programs can lead to ignoring or exacerbating existing health inequities and gender inequities or even creating new ones	The use of digital technologies in health is improving and saving lives in low and middle income countries gender and power analyses are essential digital health can be used to strengthen upward and downward accountability	The challenge is finding the balance of theoretical grounding and grounded intervention to reduce health inequities.
Szymczak *et al.* (2023) (Szymczak *et al.*, 2023)	USA	Health equity as where every person has the opportunity to attain their full health potential	Historically marginalized communities	Perspective	TM	Innovations in service delivery can inadvertently maintain, worse, or introduce inequities Telemedicine may worsen health equity by altering access to care and by altering quality of care one it is accessed Innovation technologies may obscure, deepen and facilitate oppression against historically marginalized communities	Telemedicine may improve health equity by altering access to care and by altering quality of care one it is accessed	To center equity throughout all phases of design and implementation of Telemedicine
Westby *et al.* (2021) (Westby *et al.*., 2021)	Minneapolis	Social determinants of health inequities	Older adults Under resourced populations Under-served populations	Commentary	T	Underserved populations have known barriers to face-to-face telehealth visits: lack of access to video equipment, lack of reliable internet, concerns about cost of copays, concern about cost of wireless data consumption, unfamiliarity with virtual platforms confidentiality, and trust concerns, and language barriers	Telehealth increases opportunities to reach communities and populations that lacked access to high quality health care During COVID-19 outbreak, telehealth helped to continue caring for populations while limiting risks of exposure to the virus for health care providers and patients	To improve virtual visit technology with a focus on patient ease of use, strategies to increase access to video visit equipment, universal broadband wireless and inclusion of telephone visits in reimbursement criteria for tele health
Wood *et al.* (2021) (Wood *et al.*, 2021)	USA	Digital divide as unequal access to or ability to engage in care using technological means	Vulnerable persons People who had to live in designated rural and medically underserved areas	Review	T TM EHR Infectious diseases and Human immunodeficiency virus	Digital divide Age and social isolation	-	Three requirements to benefit from digital healthcare: 1) Technology, 2) Technical literacy 3) Broadband internet connectivity Broad scale intervention from various stakeholders that would help move towards digital health equity in all fields at a national, state and local levels to address health equity and mitigate the impact of the digital divide on health outcomes for all patients

## Data Availability

The data and supportive information are available within the article.

## ABBREVIATION

DHE: Digital Health Equity

## SUPPLEMENTARY MATERIAL

PRISMA checklist is available as supplementary material on the publisher’s website along with the published article.
